# Antiparasitic and Cytotoxic Activity of Bokkosin, A Novel Diterpene-Substituted Chromanyl Benzoquinone From *Calliandra portoricensis*

**DOI:** 10.3389/fchem.2020.574103

**Published:** 2020-11-17

**Authors:** John B. Nvau, Samya Alenezi, Marzuq A. Ungogo, Ibrahim A. M. Alfayez, Manal J. Natto, Alexander I. Gray, Valerie A. Ferro, Dave G. Watson, Harry P. de Koning, John O. Igoli

**Affiliations:** ^1^Department of Chemistry, Plateau State University, Bokkos, Nigeria; ^2^Strathclyde Institute of Pharmacy and Biomedical Sciences, University of Strathclyde, Glasgow, United Kingdom; ^3^College of Medical, Veterinary and Life Sciences, Institute of Infection, Immunity and Inflammation, University of Glasgow, Glasgow, United Kingdom; ^4^Department of Veterinary Pharmacology and Toxicology, Ahmadu Bello University, Zaria, Nigeria; ^5^Department of Chemistry, Phytochemistry Research Group, University of Agriculture, Makurdi, Nigeria

**Keywords:** diterpene, trypanosomiasis, leishmaniasis, chroman-4-one, cytotocixicity

## Abstract

*Calliandra portoricensis* is a medicinal plant growing freely in Nigeria. It is used traditionally to treat tuberculosis, as an anthelmintic and an abortifacient. Phytochemical fractionation and screening of its root extracts has yielded a novel (5-hydroxy-7-methoxy-4-oxo-1-chromanyl)-4-methoxy-p-benzoquinone (breverin)-substituted cassane diterpene, which was designated bokkosin. It was obtained from column chromatography of the ethyl acetate extract of the roots. The compound was characterized using IR, NMR (1D and 2D) and mass spectral data. Promising antiparasitic activity was observed against the kinetoplastid parasite *Trypanosoma brucei brucei*, as well as moderate activity against *Trypanosoma congolense* and *Leishmania mexicana* and low toxicity in mammalian cells, with the best *in vitro* EC_50_ values against *T. b. brucei* (0.69 μg/mL against a standard laboratory strain, and its multi-drug resistant clone (0.33 μg/mL). The effect on *T. b. brucei* in culture was rapid and dose-dependent, leading to apparently irreversible growth arrest and cell death after an exposure of just 2 h at 2 × or 4 × EC_50_. The identification of bokkosin constitutes the first isolation of this class of compound from any natural source and establishes the compound as a potential trypanocide that, considering its novelty, should now be tested for activity against other microorganisms as well.

## Introduction

*Calliandra portoricensis* Benth (*Fabaceae*) is a shrub or small tree that can grow to about six meters tall. *C. portoricensis* is one of the plants used by Nigerian traditional medical practitioners to treat various infections including tuberculosis. It is used as a laxative/worm expeller (Falode et al., 2018) and an abortifacient in humans (Ayensu, 1978). The plant is distinctly ornamental, with white hemispherical flower heads and fern-like foliage. In Ghana, the root is mixed with ginger and pepper for use as an enema for lumbago pain and constipation, and to treat gonorrhea. The plant has also been reported for its anticonvulsant, analgesic, antidiarrheal, antispasmodic, antipyretic, anti-snake venom, and antirheumatic properties (Adesina, 1982; Akah and Nwaiwu, 1988; Agunu et al., 2005). In addition, it has exhibited anticholinergic, antacid, antiulcer, molluscicidal, and ovicidal activities in laboratory animals (Aguwal and Lawal, 1988), as well as activity on cancer cells (Ogbole et al., 2017; Oyebode et al., 2019). The plant extracts have been reported to possess antimicrobial activity against the following pathogens: *Escherichia coli, Staphylococcus aureus, Streptococcus faecuim* and *Candida albicans, Enterococcus faecalis, and Streptococcus pneumonia* (Adesina, 1982; Agunu et al., 2005; Enwuru et al., 2017). A pre-purified peptide fraction from *C. portoricensis* also showed promising anti-bacterial efficacy, including to methicillin-resistant *S. aureus* (Ogbole et al., 2020). However, although the plant clearly has medicinal properties, it has not yet been investigated for antiparasitic activity, and active agents have yet to be identified from the extracts.

Kinetoplastid protozoan parasites cause some of the world's most neglected infectious diseases, trypanosomiasis, and leishmaniasis. Different species of *Trypanosoma* are responsible for debilitating diseases of humans (Human African trypanosomiasis, HAT) and animals (Animal African trypanosomiasis, AAT), transmitted by the tsetse fly, mainly in tropical Africa, as well as Chagas disease, which affects large numbers of people in South and Central America and is transmitted by reduviid bugs (Barrett et al., 2003; Pérez-Molina and Molina, 2018). Tsetse-borne trypanosomiases are endemic in 37 out of 54 African countries, with serious economic and public health consequences (Yaro et al., 2016; Büscher et al., 2017). Although significant progress is being made toward the elimination of HAT as a major public health problem in Western Africa (Franco et al., 2018; Akazue et al., 2019), tens of millions of cattle, sheep, and goats as well as millions of donkeys, camels and horses are at risk of AAT (Giordani et al., 2016) including in Central and Northern Nigeria (de Gier et al., 2020). Leishmaniasis, on the other hand, is caused by at least 20 species of the genus *Leishmania*, and is endemic in about 100 countries, mostly in the tropics and subtropics, with an estimated 0.7–1 million cases annually; the disease is transmitted by phlebotomine sandflies and consists of cutaneous, mucosal and visceral clinical forms depending on the species (Burza et al., 2018).

Chemotherapy for both trypanosomiasis and leishmaniasis is based on old drugs and is associated with a myriad of challenges such as drug toxicity, chemoresistance, and a lack of guaranteed supply (Delespaux and De Koning, 2007; Burza et al., 2018; Altamura et al., 2020; de Koning, 2020). Therefore, there is an urgent need for new treatment approaches. Nevertheless, the progress in discovering new and effective antiparasitic drugs has been very poor. Possible reasons for this include an over confidence in the validation of single molecular targets such as enzymes or receptors and a lack of fundamental knowledge on the biology and metabolism of these kinetoplastids (Field et al., 2017). An alternative approach to high throughput screening against a single target protein, which may accelerate the discovery of novel leads, is to use high throughput phenotypic screening assays against live parasites *in vitro* (Nagle et al., 2014; Peña et al., 2015). Such phenotypic screening may be more time effective in lead generation, as it has the advantage of identifying compounds that are able to cross cell membranes and additionally, identify active compounds with unknown and/or multiple targets. Such compounds might not be identified in a screen with a defined single target such as an enzyme (Field et al., 2017), sometimes because they accumulate strongly in a particular cellular compartment (Lüscher et al., 2007), e.g. the mitochondrion (Alkhaldi et al., 2016; Fueyo González et al., 2017).

Nature has been a rich source of biologically active compounds for the development of new pharmacological agents themselves or from which active compounds have been derived using their novel structures as a template, over the past decades (Cragg and Newman, 2005; Newman and Cragg, 2020). Several compounds isolated from natural sources have been shown to inhibit the growth of trypanosomes *in vitro* and *in vivo*, e.g., cordycepin and its chemical analogs (Vodnala et al., 2013; Hulpia et al., 2018, 2019a), isolated from the fungus *Cordyceps militaris*, and quercetin and its derivatives, which is a polyphenolic flavonoid commonly found in plants (Mamani-Matsuda et al., 2004). Other examples include the trypanocidal activity of two dipeptide compounds isolated from the roots of *Zapoteca portoricensis* (Nwodo et al., 2014), a clerodane-type diterpenoid isolated from *Polyalthia longifolia* (Ebiloma et al., 2018a), steroid alkaloids from *Holarrhena africana* (Nnadi et al., 2019) and the anti-kinetoplastid properties of propolis (Alotaibi et al., 2019; Siheri et al., 2019).

In this study, we report the isolation of a novel breverin-substituted cassane diterpene, designated bokkosin, from the Nigerian indigenous medicinal plant *C. portoricensis*, and examined its *in vitro* activity against kinetoplastid parasites and its toxicity to mammalian cell lines.

## Materials and Methods

### Chemical Extraction and Characterization

#### Plant Material

The roots of *C. portoricensis* were purchased from a traditional healer in Jos market, Plateau State, Nigeria, certified at the Department of Forestry Technology, Federal School of Forestry, Jos, Nigeria, and a voucher specimen with herbarium number NIPRD/H/6244 was deposited at the National Institute for Pharmaceutical Research and Development (NIPRD) Abuja, Nigeria. The roots were dried at room temperature for 2 weeks, before being ground into a powder and used for extraction.

#### Equipment

The IR spectrum was acquired on a Shimadzu IRAffinity-1 spectrophotometer while the ^1^H and ^13^C NMR spectra were run on a Bruker AVIII (400 MHz) spectrophotometer (^1^H, 100 MHz; ^13^C, 400 MHz) using deuterated benzene C_6_D_6_ as the solvent. The LC-HRMS was run on a Bruker Compass LC-MS spectrometer. Column chromatographic separations were carried out in glass columns using silica gel (200–400 mesh) and spots on thin layer chromatography [TLC grade Silica gel 60 F_253_, Merck KGaA (Darmstadt, 64293, Germany)] were visualized using ethanol:sulfuric acid (90:10) reagent. Solvents for extraction and column chromatography were purchased from Sigma Aldrich, United Kingdom.

#### Extraction

The powdered root (1.50) kg was successively extracted using cold maceration with 3 liters of hexane and ethyl acetate (HPLC grade, Sigma Aldrich, United Kingdom) for 72 h each. The extracts were concentrated on a rotatory evaporator at 40°C under vacuum and subsequently evaporated to dryness on a water bath to obtain 18.7 g of hexane (12.5%) and 34.5 g of ethyl acetate (23.0%) extracts. The ethyl acetate extract (1.20 g) was dissolved in ethyl acetate and adsorbed on silica gel (200–400 mesh, 10 g, Merck Germany,). The slurry was allowed to dry at room temperature and a dark-brown powder was obtained. A glass column (diameter 6 × 50 cm height) was wet-packed with silica gel (200–400 mesh, 100 g) using hexane as the solvent. The adsorbed extract was loaded onto the column and eluted with 300 mL each of increasing ratios of ethyl acetate in hexane: 100:0, 90:10, 80:20, 70:30, 60:40, 50:50, 40:60, 30:70, 20:80, 10:90, 0:100, with collection of 20 mL fractions. The fractions were monitored by TLC on pre-coated aluminum sheets coated with silica gel F250 (Merck, Germany) and on that basis grouped into eight combined fractions (F1 to F8). Fraction F3 (30% ethyl acetate) was subjected to further column chromatography. Another column was similarly packed with 50 g of silica gel and eluted using increasing amounts of ethyl acetate in hexane, resulting in the isolation of compound **1**.

### Determination of Biological Activity

#### Parasites, Mammalian Cell Lines, and Culture

The kinetoplastid parasites used in this study included the bloodstream form (BSF) of *T. b. brucei* s427WT and the derived drug resistant clone *B48* that lacks both the TbAT1/P2 transporter and the high affinity pentamidine transporter (HAPT1) and is therefore highly resistant to diamidine and melaminophenyl arsenical drugs (Bridges et al., 2007). For *T. congolense* BSF IL3000WT and its 6C3 diminazene resistant clone, generated through *in vitro* drug exposure, were used (Alotaibi et al., 2019). As a sample *Leishmania* species, *L. mexicana* cas9/T7 (Beneke et al., 2017) and the derived *L. mexicana* cas9^Δ*NT*1^ promastigotes were used. The *T. b. brucei* strains were cultured at 37°C in a 5% CO_2_ atmosphere, in HMI-9 medium (Invitrogen, UK) supplemented with 10% (v/v) heat inactivated fetal bovine serum (FBS), 14 μL/L β-mercaptoethanol, and 3.0 g/L NaHCO_3_ adjusted to pH 7.4 as described previously (de Koning et al., 2000). *T. congolense* strains were cultured at 34°C/5% CO_2_, essentially as described previously (Coustou et al., 2010; Ebiloma et al., 2018b) in basal medium prepared with MEM medium (Sigma-Aldrich), 26 mM NaHCO_3_, 25 mM HEPES, 5.6 mM D-glucose, 1 mM sodium pyruvate, 100 μM hypoxanthine, 40 μM adenosine, 16.5 μM thymidine, and 25 μM bathocuproine disulfonic acid disodium salt, and supplemented with 1.6 mM glutamine, 100 units/mL penicillin, 0.1 mg/mL streptomycin, β-mercaptoethanol (0.0014%, v/v), 15% (v/v) goat serum (Gibco, United Kingdom), and 5% (v/v) Serum Plus II (Sigma-Aldrich, United Kingdom). *L. mexicana* strains were cultured at 25 °C and 5% CO_2_ in HOMEM medium supplemented with 10% (v/v) FBS and 1% (v/v) penicillin/streptomycin solution (Gibco, United Kingdom) (Al-Salabi et al., 2003; Khandazhinskaya et al., 2019). Human U937 cells (European Collection of Cell Cultures Cat. No. 85011440, supplied by Sigma-Aldrich, United Kingdom), were cultured in RPMI 1640 medium (Lonza, United Kingdom) supplemented with 5% (v/v) FBS, 1% (v/v) L-glutamine and 1% (v/v) penicillin/streptomycin at 37°C in a humidified atmosphere of 5% CO_2_ as previously described (Passmore et al., 2001). RAW 264.7 (ATCC TIB-71, United States) murine macrophages were cultured using ATCC-formulated Dulbecco's Modified Eagle's Medium (DMEM) (Catalog No. 30-2002) supplemented with 10% (v/v) FBS, 1% (v/v) glutamine, and 1% (v/v) penicillin-streptomycin at 37°C in a humidified atmosphere of 5% CO_2_ (Han et al., 2002).

#### Construction of the Derived *L. Mexicana* cas9^ΔNT1^ Cell Line

The closely related genes *LmexNT1.1* and *LmexNT1.2* transport adenosine, thymidine, and uridine (Alzahrani et al., 2017; Campagnaro and De Koning, 2020) as well as a number of purine and pyrimidine nucleoside analogs with strong antileishmanial activity, including the antibiotic tubercidin (Vasudevan et al., 1998; Aoki et al., 2009), and 5-F-2′-deoxyuridine (Alzahrani et al., 2017). Since these genes are functionally highly conserved in *Leishmania* species (Alzahrani et al., 2017) and tubercidin analogs have recently been reported to have excellent anti-kinetoplastid activity *in vitro* and *in vivo* (Hulpia et al., 2018, 2019a,b; Lin et al., 2019) this strain was included here as a further control. The knockout strategy for the NT1.1/NT1.2 allele in *L. mexicana* cas9 was performed as described previously (Beneke et al., 2017). Briefly, primers 1 to 5 (Supplementary Table 1) were used to produce NT1 KO in *L. mexicana* cas9 and these primers were designed using the LeishGEdit online platform (Beneke et al., 2017). The deletion of the *NT1* locus requires the amplification of two sgRNAs, using primers 1–3 to direct Cas9 to cut immediately upstream (5′) or downstream (3′) of the target locus of *NT1*. To create CRISPR plasmids specific to the target locus of *NT1*, pTBlast, and pTPuro plasmids (with blasticidin and puromycin cassettes, respectively), were amplified using primers 4 and 5. These constructs were to integrate into the *NT1* locus after cutting with the two sgRNAs. Thus, to yield the NT1 KO, *L. mexicana-*cas9 was transfected once, with two sgRNA templates and two antibiotic resistance markers (pTBlast and pTPuro). The combination of the two antibiotic resistance markers was used to create homozygous cells that do not retain a copy of *NT1*. The loss of the *NT1* locus in drug-resistant transfectants was verified by performing PCR diagnostics to amplify PCR products within the open reading frame of the *NT1* locus, using primers that are unique to the *NT1* locus. A region of the *NT1* amplicon was amplified with genomic DNA-specific primers (forward primer, 5′- TCCGCTGCAAACAAACTTCTGG-3′; reverse primer, 5′-TACGCCGCTACGATGATCCAGC-3′) and PCR products were run on 1% agarose gel to be observed visually. PCR diagnostics of the *L. mexicana* cas9 and *L. mexicana* cas9^Δ*NT*1^ cell lines showed the expected presence and absence of *NT1*-specific bands in the parental cell line and the KO clone, respectively (~1 kb) (Supplementary Figure 1).

#### Determination of the Cytotoxic Effect of Bokkosin on U937 and RAW 246.7 Mammalian Cell Lines

The cytotoxic effect of bokkosin on RAW246.7 was carried out as previously described (Han et al., 2002; Ayupova et al., 2019). Cells were grown to log phase and adjusted to a density of 1 × 10^5^ cells/ml. One hundred microlitre/well of the cells were added to a 96 well plate (TPP, Switzerland) and the plate incubated for 24 h at 37°C, 5% CO_2_, 100% humidity. The stock solution was prepared in another 96 well plate in 8 different concentrations in the full, supplemented DMEM medium (section Parasites, mammalian cell lines, and culture) using 1:1 serial dilution (i.e., starting from 200 μg/mL as the top concentration until 1.56 μg/mL). The samples were then transferred (100 μL) to the cultured cells using a multichannel pipette and left in the incubator for 24 h. After incubation, 5 mM resazurin sodium salt (Sigma Aldrich, UK) was added (20 μL per well) and the plate incubated for a further 24 h. Fluorescence readings of the plate were taken using a Perkin Elmer Wallac Victor2 microplate reader (λex/em: 544 and 590 nm). While, U937 cells were grown to log phase, counted and adjusted to a density of 1 × 10^5^ cells/mL. A volume of 100 μL of cells was then added to each well of a 96-well plate (TPP, Switzerland) and incubated for 24 h at 37 °C, 5% CO_2_ and 100% humidity. From a 200 μg/mL stock solution, a 2-fold serial dilution of the test compound was carried out in the full, supplemented RPMI medium, (section Parasites, mammalian cell lines, and culture) to determine the EC_50_ value for the samples. In another 96-well plate, 100 μL of each dilution was transferred to the cultured cells using a multichannel pipette, followed by incubation for 24 h. Controls consisted of: 10% (v/v) DMSO (positive, cell death control), cells and medium (negative control) and 0.5% (v/v) DMSO (solvent control). This was followed by addition of 20 μL of 5 mM resazurin sodium salt (Sigma Aldrich, United kingdom) and the plates incubated for a further 24 h after which fluorescence was measured using a Wallac Victor 2 (Perkin Elmer, United Kingdom) microplate reader (λ_exc_ = 560 nM, λ_em_ = 590 nm). The compound was tested in triplicate and cell viability was expressed as a percentage of the drug-free control. The resulting data were analyzed using GraphPad Prism 5.0 (GraphPad Software Inc., San Diego) to obtain a dose-response curve and the mean 50% effective concentration (EC_50_) value.

#### Determination of *in vitro* Anti-trypanosomal and Anti-leishmanial Activity of Bokkosin

Bokkosin was tested against BSF *T. b. brucei* s427 WT and *T. b. brucei* B48; BSF *T. congolense Tc*-IL3000 WT and *T. congolense* 6C3; and *L. mexicana* cas9/T7 and *L. mexicana* cas9^Δ*NT*1^ promastigotes in order to assess its antiparasitic activity and the potential for cross-resistance with existing drugs using resazurin-based assays as described previously (Fueyo González et al., 2017; Eze et al., 2019; Khandazhinskaya et al., 2019). This assay is based on the fact that the blue non-fluorescent dye resazurin is metabolized by live, but not dead cells, including *Trypanosoma* and *Leishmania* species, to the red, fluorescent compound resorufin (Gould et al., 2008). Briefly, 11 double dilutions of the compound (in the appropriate culture medium for the species) were prepared from 20 mg/mL stock solution in 96-well plates (Greiner Bio-one GmbH, Germany), starting from 200 μg/mL down to 0.19 μg/mL with the 12^th^ well containing no drug. Dilutions of pentamidine or diminazene were prepared in parallel to serve as positive controls. The cells were then adjusted to twice the required density in the appropriate medium and added to the wells containing drug dilutions followed by incubation for 48 or 72 h, as shown in Table 1, after which 20 μL of 125 μg/mL resazurin sodium salt (Sigma Aldrich, United Kingdom) in phosphate-buffered saline (PBS, Sigma Aldrich, UK) was added. The plates were further incubated for 24 or 48 h under the same conditions and fluorescence was determined using a FLUOstar Optima plate reader (BMG Labtech, United States), λ_exc_ = 544 nM, λ_em_ = 590 nm. The results were expressed as half maximal effective concentration (EC_50_) values, which were calculated by non-linear regression using an equation for a sigmoidal dose-response curve with variable slope (GraphPad Prism 5.0).

**Table 1:** Seeding density, culture conditions, and incubation period for resazurin-based assays in different parasite cell lines.

**Cell line**	**Seeding density**	**Condition**	**Incubation time (h)**	
	**(cells/well)**		**Initial**	**+ resazurin**
*T. b. brucei* 427WT and B48	2 × 10^4^	37°C, 5% CO_2_	48	24
*T. congolense Tc*-IL3000 WT and *T. congolense* 6C3	5 × 10^4^	34°C, 5% CO_2_	48	24
*L. mexicana* cas9/T7 and cas9^Δ*NT*1^	4 × 10^5^	27°C	72	48

#### Effect of Bokkosin on the Growth of *T. b. brucei* s427 Wild Type

The effect of different concentrations of bokkosin, on the growth of *T. b. brucei* s427 wild type *in vitro* was tested following continuous exposure over 48 h, as described previously (Hulpia et al., 2019a). Briefly, the cells were counted and adjusted to a density of 2 × 10^5^ cells/mL in 5 mL of either fresh complete HMI-9 medium or in medium containing bokkosin at 0.5×, 1×, 2×, and 4× EC_50_ (determined by the resazurin-based assay, above) in 25-mL culture flasks. The flasks were incubated at 37°C and 5% CO_2_ and the cells of each culture were counted in a Neubauer counting chamber under a phase-contrast microscope in triplicate at several time points (0, 2, 6, 12, 18, 24, 30, 36, 42, and 48 h) for each concentration of the compound, as well as the no drug control. An additional experiment, using a wash-out of the test compound at just 2 h was performed to investigate whether limited exposure time would be sufficient for irreversible impact on cell growth. In this case, cells were washed after the 2-h drug incubation, by centrifugation at 1,600 × *g*, followed by reconstitution in the same volume of fresh media. The growth experiments were performed on three separate occasions and the mean used to plot the growth curve.

## Results and Discussion

### Characterization of Compound 1 (Bokkosin) as a (5-hydroxy-7-methoxy-4-oxo-1-chromanyl)-4-methoxy-p-benzoquinone (breverin)-Substituted Cassane Diterpene

**Figure 1:**
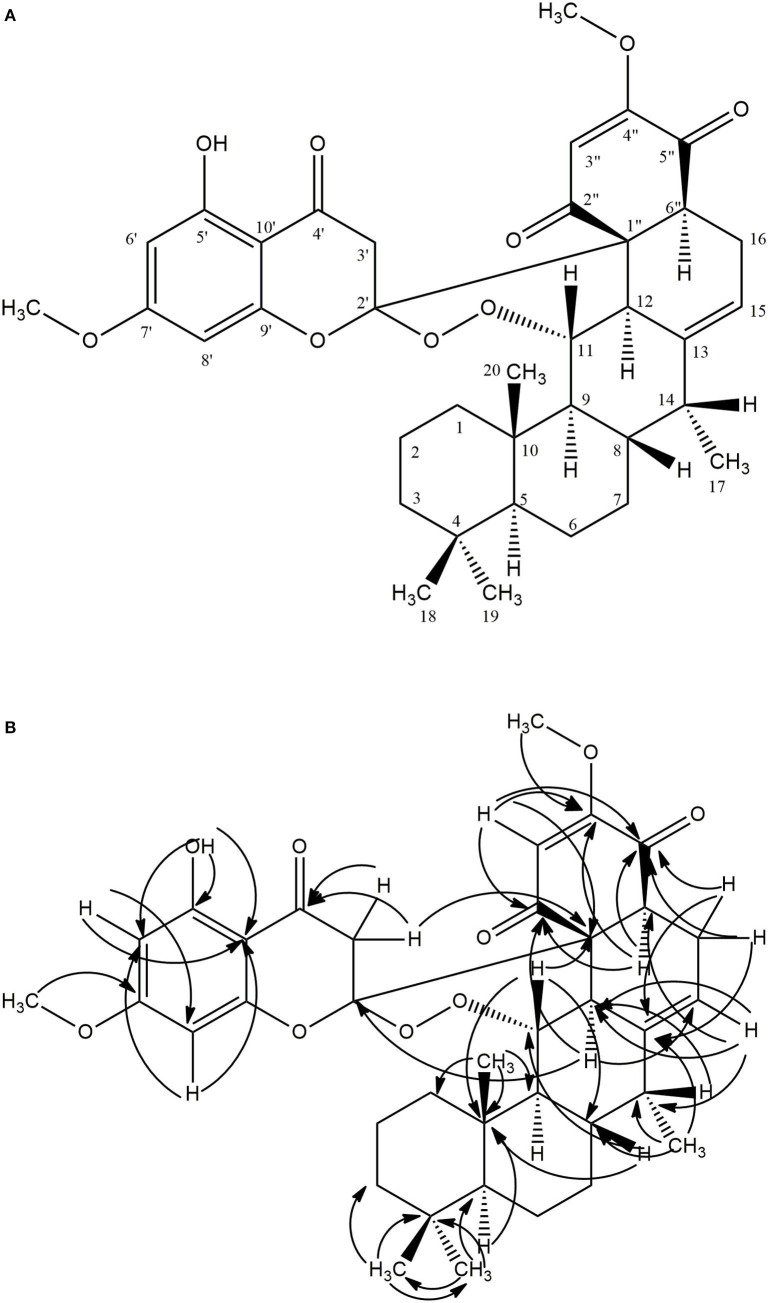
Structure of bokkosin and its 2D correlations. **(A)** COZY (Dark lines), NOESY (Arrows), **(B)** HMBC (Arrows).

Compound **1** (80.0 mg) was obtained as a white crystalline solid (mp 290–301°C). The LC-MS spectrum (Supplementary Material 1) gave a [M+H]^+^ ion at m/z = 633.4234 (calcd 633.3064, C_37_H_45_O_9_) corresponding to the molecular formula C_37_H_44_O_9_, and having 16 units of unsaturation. The IR spectrum (Supplementary Material 2) showed absorption frequencies for ketone carbonyls (C=O) between 1,700 and 1,750 cm^−1^, C-H at 2,900, C-O at 1,200 and 1,250, aromatic and alkene C=C double bonds in the region of 1,450–1,600 and aromatic substitution between 800 and 900 cm^−1^. The proton spectrum (Supplementary Material 3) showed a deshielded proton signal for a hydrogen bonded hydroxyl group at δ_H_ 12.30 typical of a 5-hydroxy flavanone moiety. There were signals corresponding to six methyl groups made up of three aliphatic singlets at δ_H_ 0.69, 0.74, 0.79 (3H, s), one doublet at 1.00 (3H, d, *J* = 7.1 Hz) and two highly shielded methoxy groups at 2.82 and 3.03 (3H, s). It also showed eleven methine signals made up of seven aliphatic methine protons at δ 0.63, 1.34, 1.35, 2.01, 3.48, 3.58, 3.66, two aromatic methine protons at 6.02 and 6.11, and two olefinic protons at 5.09 and 5.62 ppm. The spectrum also had signals for seven pairs of methylene protons. The ^13^C NMR spectrum (Supplementary Material 4) showed signals for 37 carbon atoms consisting of three ketone carbonyls at δ_C_ 192.3, 193.3, 194.1, five quaternary aromatic carbons at 102.5, 158.8, 164.0 (–OH substituted), 161.1 and 167.7 (-OCH_3_ substituted), 141.2 for an olefinic quaternary, and 110.6 for a quaternary acetal carbon. There were signals for six methyl carbons at δ_C_ 11.4, 14.6, 21.3, 33.6,54.7, 55.3, the latter two being methoxys, three oxymethines at δ_C_ 54.7, 55.3, 82.5 ppm and seven methylene carbons at 18.8, 21.6, 29.2, 31.2, 40.4, 41.7, and 45.8 ppm. It also showed eleven methine carbons consisting of two aromatic ones at 94.4 and 95.4, two olefinic methines at 111.1 and 113.1 and six aliphatic ones at 39.6, 41.6, 44.4, 45.4, 52.7, 54.2 ppm. Using the 2D NMR (COZY, HSQC, HMBC, Supplementary Materials 5–7) spectra for the compound, the structure (Figure 1) was established as follows: the two aromatic protons at δ 6.02 (d, *J* = 2.4 Hz) and 6.11 (d, *J* = 2.4 Hz) were meta coupled and confirmed the substitution in ring A of the flavanone part with strong HMBC correlations to one another's carbons and to C-7′ and C-10′. Long range correlations from the chelated –OH proton at 12.30 ppm to C-5′, C-6′, and C-10′ confirmed the –OH to be at C-5′ and the carbonyl at C-4′. The chemical shift of the C-4′ carbonyl was at 193.3 ppm indicating a saturated ring ketone, confirmed by methylene proton (H-3′) correlations to it and to C-2′ (a quartenary carbon with chemical shift (δ 110.6) similar to that of a di-oxygenated acetal-type carbon). One of the H-3′ protons (δ_H_ 2.88) also correlated with C-10′. The methylene protons both correlated with another quaternary carbon (δ 62.3) which was not part of the chromanone ring system and must be C-1″. This carbon also had correlations from protons at δ_H_ 5.62 and 3.66 and both of these protons, H-3″ and H-6″, respectively, correlated with two carbonyls (C-2″ & C-5″) and a methoxy-bearing carbon (C-4″, δ_C_ 161.4) completing a six-membered quinonoid ring-B of the flavonoid part of the molecule. This accounts for 17 carbons in total for the flavanone portion of the molecule bearing two methoxy groups. There are now 20 carbons in the rest of the molecule including four methyl groups and one double bond and the proton spectrum (Supplementary Material 2) was typical of a diterpene-type moiety. This was confirmed in the HMBC (Supplementary Material 7) by long range correlations from two geminal methyl groups H-18 and H-19, identifying C-3, C-4, C-5, and their carbons C-18 and C-19. Further, correlations from the H-17 doublet to C-13 indicated a double bond between C-13 and C-15. Correlations from H-15 to C-12 and C-16 and from H-20 to C-1, C-5, C-9 and C-10 confirmed the diterpene to be a cassane type skeleton. The cassane moiety must be attached to the flavanoid B-ring via the connections C-12 to C-1″ and C-16 to C-6″ as there were COZY (Supplementary Material 5) correlations between H-16 and H-6″ and HMBC (S7) correlations between H-12 and the quinonoid carbonyl (δ_C_ 194.1) C-2″ and H-16 with C-1″ and C-5″. There was even a significant long range zig-zag ^4^*J* correlation in the HMBC spectrum between H-3″ and C-12. Taking all of the atoms from both flavonoid and diterpenoid moieties into account, there remained two oxygens unaccounted for in the above arguments. These must be attached as a peroxide bridge between C-11 and C-2′ and the chemical shifts of these two carbons (82.5 and 110.6 ppm, respectively) testify to this proposition. The NOESY spectrum (Supplementary Material 8) revealed the strong correlations between the methoxys and their *ortho*-proton neighbors, established the relative stereochemistry within the cassane moiety and between cassane H-12 (δ_H_ 3.48) and the flavanone H-3′ (δ_H_ 2.88) (Figure 1). Thus, the compound was characterized as a novel 2-(5-hydroxy-7-methoxy-4-oxo-2-chromanyl)-5-methoxy-p-benzoquinone (breverin) (Allan et al., 1973) substituted at C-12 and C-16 of a cassane diterpene with a peroxide bridge between C-11 of the diterpene and C-2 of the breverin part (Figure 1) and named bokkosin. The ^1^H and ^13^C chemical shift assignments in C_6_D_6_ are given in Table 2.

**Table 2:** ^1^H and ^13^C chemical shifts of compound 1 in C_6_D_6_.

**Position**	**Chemical shift**		**HMBC**
	^1^H [δ ppm, mult, *J* (Hz)]	^13^C (δ ppm, mult)	
1	0.92 (m), 1.84 (dq, 13.6, 2.8)	40.4 (CH_2_)	C-3,C-5,C-20
2	1.15, 1.25(m)	18.5 (CH_2_)	C-4, C-10
3	1.03, 1.18 (m)	41.7 (CH_2_)	C-7, C-19, C-20
4	–	32.9 (C)	
5	0.63 (dd, 2.0, 2.2)	54.2 (CH)	C-7, C-19, C-20
6	1.45 (m), 1.02 (m)	21.6 (CH_2_)	C-8, C-10
7	1.13 (m), 1.32 (m)	31.2 (CH_2_)	C-5,C-14
8	1.35 (m)	39.6 (CH)	C-11,C-13, C-17
9	1.34 (m)	52.7 (CH)	C-10, C-14
10		37.7 (C)	
11	3.57 (m)	82.5 (CH)	C-9, C-12, C-1″
12	3.48 (d, 11.3)	44.4 (CH)	C-9, C11, C-2″
13		141.2 (C)	
14	2.01(m)	41.6 (CH)	
15	5.10 (dt, 6.4, 1.8)	113.1 (CH)	C-12, C-16
16	2.12 (dt, 16.7, 5.6), 2.00 (m)	29.2 (CH_2_)	
17	1.00 (d, 7.1)	14.6 (CH_3_)	C-8, C-13, C-14
18	0.79 (s)	33.6 CH_3_)	C-3, C-4, C-5, C-19
19	0.69 (s)	21.3 CH_3_)	C-3, C-4, C-5, C-18
20	0.74	11.4 CH_3_)	C-1, C-5, C-9, C-10
1′			
2′		110.6 (C)	
3′	2.90, 2.88 (ABq,16.1)	45.8 (CH_2_)	C-2′, C-10′, C-1″
4′		193.3 (C)	
5′		164.0 (C)	
6′	6.02 (d, 2.4)	94.4 (CH)	C-7′, C-8′, C-10′
7′		167.7 (C)	
8′	6.11 (d, 2.3)	95.4 (CH)	C-6′, C-7′, C-10′
9′		158.8 (C)	
10′		102.5 (C)	
1″		62.3 (C)	
2″		194.1 (C)	
3″	5.62 (s)	111.1 (CH)	C-2″, C-3″, C-4″
4″		161.4 (C)	
5″		192.3 (C)	
6″	3.66 (dd, 12.2, 5.0)	45.4 (CH)	C-16, C-2′, C-1″,C-2″, C-4″, C-5″
5′-OH	12.30 (s)		C-5′, C-6′, C-10′
7′-OCH_3_	3.03 (s)	54.7 (CH_3_)	C-7′
4″-OCH_3_	2.85 (s)	55.3 (CH_3_)	C-4″

### *In vitro* Antitrypanosomal and Antileishmanial Activity and Cross Resistance Studies of Bokkosin

The *in vitro* activity of bokkosin on wild-type *T. b. brucei* s427 and the derived, multi-drug resistant clone B48 (Munday et al., 2014) was determined in three independent resazurin-based drug sensitivity assays as shown in Table 3. Bokkosin displayed potent activity against *T. b. brucei* s427 with an EC_50_ value of 0.69 ± 0.04 μg/mL, corresponding to approximately 1 μM, and was not cross resistant to pentamidine. In fact, the B48 clone was significantly more sensitive to bokkosin (*P* = 0.0017; Student's unpaired, two tailed *t*-test) with a Resistance Factor (RF) of 0.49 compared to 159-fold resistance for pentamidine (*P* < 0.0001). Given the highly promising activity observed against *T. b. brucei* s427 and *T. b. brucei* B48, it was decided to determine the activity of compound **1** against *T. congolense*, which is the most important etiological agent of AAT (Giordani et al., 2016). Although not as active as against *T. b. brucei*, bokkosin showed modest activity against *T. congolense* IL3000 WT (Table 4) but the EC_50_ of 21.6 μM does not justify further development against this parasite. No cross-resistance to the widely used drug diminazene aceturate was observed (RF relative to *T*. *congolense* 6C3 was 0.81; *P*>0.05) as shown in Table 4. Interestingly, bokkosin was somewhat more active against the diamidine-resistant strains of *T. b. brucei* and *T. congolense* than against the sensitive controls, although this was only statistically significant for *brucei*. It is difficult to speculate at this point as to the reasons for this. However, it is known that the diamidine transporters of both trypanosome species are completely different, and thus those drug transporters are highly unlikely to be involved in the uptake of bokkosin; however, in both species these cationic diamidines target the mitochondrion aided by the mitochondrial membrane potential, which is reduced in *T. congolense* 6C3 (Carruthers et al., 2020). A possible interpretation of these observations would be that the target for bokkosin is not mitochondrial. However, this is a new compound and as such the mechanism of its trypanocidal action is a matter of speculation at this moment.

**Table 3:** EC_50_ of bokkosin on *T. b. brucei* S427 WT and *T. brucei* B48.

**Sample**	***T. brucei*** **s427 WT**		***T. brucei*** **B48 (Pentamidine Resistant)**			
	**EC**_****50****_		**EC**_****50****_		**RF**	***t*-test**
	**(μg/mL)**	**(μM)**	**(μg/mL)**	**(μM)**		
Bokkosin	0.69 ± 0.04	1.09 ± 0.06	0.33 ± 0.05	0.53 ± 0.08	0.49	0.0017
Pentamidine		0.0034 ± 0.0004		0.55 ± 0.03	159	6.10 × 10^−5^

*RF, Resistance factor; EC_50_ (resistant strain)/EC_50_ (WT). RF ≤ 1 means no cross resistance with Pentamidine; RF significantly >1 shows cross resistance. All EC_50_ values are the average and SEM of three independent determinations. Statistical significance was determined using Student's unpaired t-test; P < 0.05 is considered significant. Molecular weight of bokkosin = 632.75 g/mol*.

**Table 4:** EC_50_ of bokkosin on *T. congolense* IL3000 WT and *T. congolense* 6C3.

**Sample**	***T. congolense*** **IL3000 WT**		***T. congolense*** **6C3 (Diminazene resistant)**			
	**EC**_****50****_		**EC**_****50****_		**RF**	***t*-test**
	**(μg/mL)**	**(μM)**	**(μg/mL)**	**(μM)**		
Bokkosin	21.6 ± 2.0	34.8 ± 3.2	17.5 ± 0.4	28.4 ± 0.6	0.81	0.11
Diminazene		0.30 ± 0.07		1.6 ± 0.3	5.4	0.015

*RF, Resistance factor; EC_50_ (resistant strain)/EC_50_ (WT). All EC_50_ values are the average and SEM of three independent determinations. Statistical significance was determined using Student's unpaired t-test; P < 0.05 is considered significant*.

Bokkosin was also tested against two cell lines of *L. mexicana*, a species that causes severe cutaneous leishmaniasis from the southern United States to the northern regions of South America (Kevric et al., 2015). CRISPR technology was used and the *L. mexicana* cas9/T7 strain (Beneke et al., 2017) to knockout both copies of the NT1.1 - NT1.2 allele from the genome. As expected, the cas9^Δ*NT*1^ strain was highly resistant to tubercidin (56.6-fold; *P* < 0.0001). Bokkosin displayed low micromolar activity against promastigotes of the cas9/T7 strain and no significantly difference against cas9^Δ*NT*1^ (*P* > 0.05), as shown in Table 5. This preliminary result against the promastigote (insect) form was not deemed to be sufficiently encouraging to continue to test against intra-macrophage amastigotes, which would be necessary for a more definitive indication of clinical potential, particularly since the activity against multidrug resistant *T. b. brucei* was ten-fold higher.

**Table 5:** EC_50_ of bokkosin on *L. mexicana* cas9/T7 and *L. mexicana* cas9^ΔNT1^.

**Sample**	***L. mexicana*** **cas9/T7**		***L. mexicana*** **cas9**^****Δ***NT***1****^			
	**EC**_****50****_		**EC**_****50****_		**RF**	***t*-test**
	**(μg/mL)**	**(μM)**	**(μg/mL)**	**(μM)**		
Bokkosin	5.8 ± 1.5	9.1 ± 2.4	9.2 ± 0.9	14.5 ± 1.4	1.59	0.12
Tuberculin		0.44 ± 0.01		24.8 ± 0.2	56.6	<0.0001
Pentamidine		0.84 ± 0.13		0.91 ± 0.02	1.08	0.65

*RF, Resistance factor; EC_*50*_ (resistant strain)/EC_*50*_ (WT). All EC_*50*_ values are the average and SEM of three independent determinations. Statistical significance was determined using Student's unpaired t-test; P < 0.05 is considered significant*.

### Effect of Bokkosin on *in vitro* Growth of *T. brucei* s427 WT BSF

**Figure 2:**
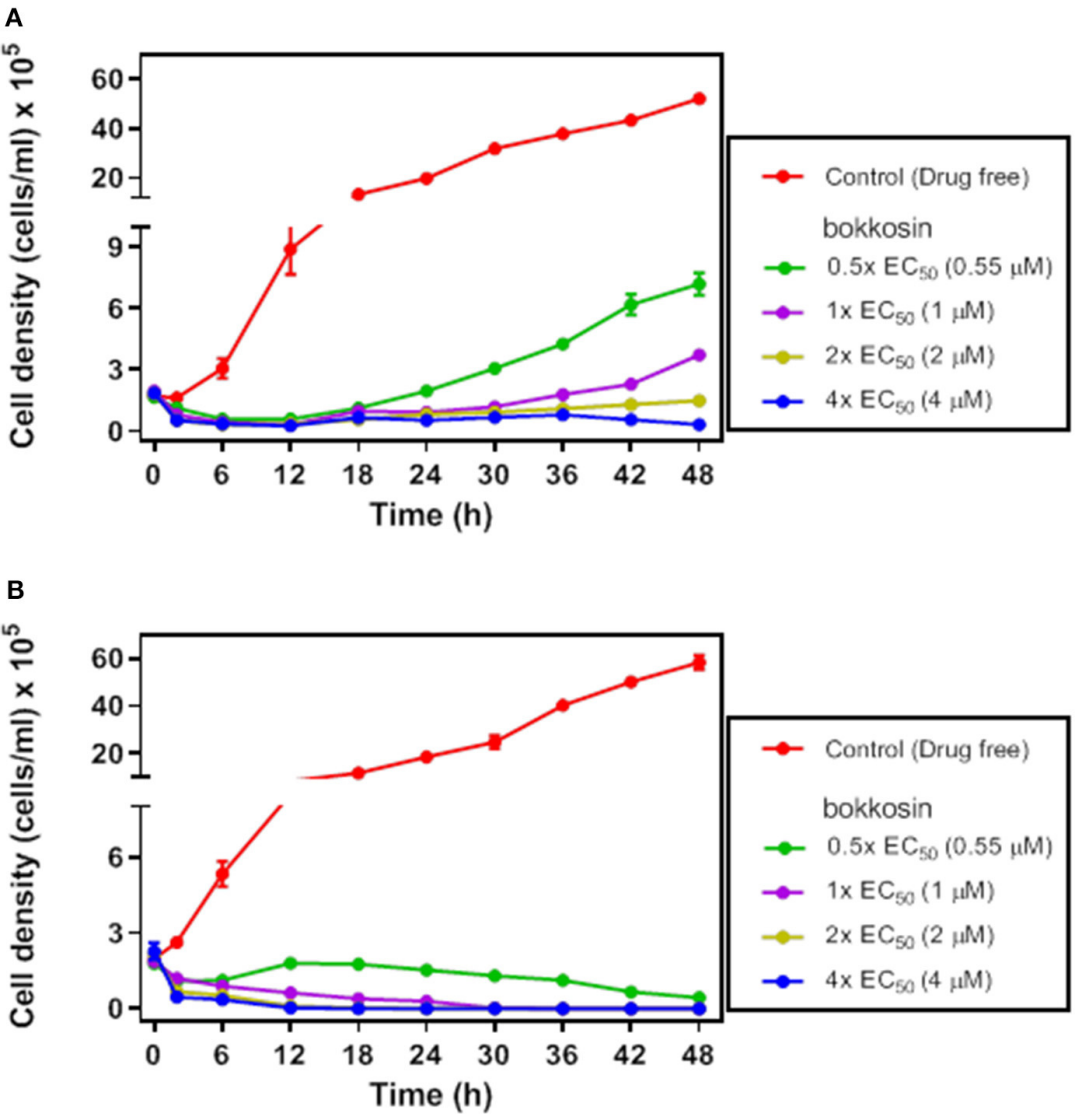
Growth of *T. brucei* s427WT in drug free culture (control) or in the presence of 0.5×, 1×, 2× or 4× the EC_50_ concentration of bokkosin. Cell seeding density was 2 × 10^5^ cells/mL in HMI-9 with 10% (v/v) FBS, incubated at 37°C and 5% CO_2_ (drug free) and counted at 0, 2, 6, 12, 18, 24, 30, 36, 42, and 48 h. Growth curves show the effect of test compounds on trypanosome growth after a limited exposure of 2 h **(A)** or continuous exposure to the drug **(B)**. Cell counts are averages of three independent experiments, each counted in triplicate.

In order to verify whether bokkosin is trypanocidal or trypanostatic, its effect was tested on log phase growth of *T. brucei* 427WT BSF culture over time. The growth curves of the parasite following a 2 h short exposure and continuous exposure to bokkosin are shown in Figures 2A,B.

The result shows that continuous exposure to bokkosin for 48 h achieves complete clearance of trypanosomes from the culture, even at half the EC_50_ concentration; no live cells were detected after exposure times of 36, 24 and 18 h, respectively for 1×, 2×, and 4× EC_50_ concentration, respectively. Following only a short exposure of 2 h, the effect appears to be a prolonged growth arrest, as cultures incubated with it at 0.5×, 1×, and 2× EC_50_ were still able to grow, albeit at a very slow rate dependent on the drug concentration. At 4× EC_50_ growth did not recover over the period of the experiment, and growth arrest appears to have been irreversible, although a definitive assessment would require an even longer incubation time. This shows that bokkosin is trypanocidal upon prolonged exposure and trypanostatic on short exposure. Generally, for the translation into *in vivo* activity, a short minimum exposure time for a drug is desirable and may reduce potential toxic effects associated with keeping compounds at peak circulation levels for longer. In this respect, it is encouraging that the compound, even at concentrations ≤ EC_50_ and submicromolar, strongly impacted cell density within the first 2 h. In addition, the practicalities of treating animal trypanosomiasis, for instance, make it highly desirable that a single administration be effective. Consequently, the strong inhibitory effect of even the limited exposure time of *T. b. brucei* to the test compound suggests that the compound is promising, at least against this trypanosome species.

### *In vitro* Cytotoxicity of Bokkosin on Mammalian Cells

In order to determine whether the activity of bokkosin is specifically antiprotozoal or the result of general toxicity, the compound was tested on U937 human cells and RAW267.4 murine cells. Bokkosin showed low toxicity to these cell lines, with EC_50_ values >200 μM, thus displaying remarkable selectivity against kinetoplastid parasites compared to the mammalian cells, as shown in Table 6. Although this did not result in high selectivity index (SI) values for *T. congolense* and *L. mexicana*, the SI for both *T. brucei* strains were >200, which should be considered encouraging for all *T. brucei*-group species, which include the animal pathogens *T. b. brucei, T. evansi, T. b. rhodesiense*, and *T. equiperdum*, as well as the causative agents of HAT, *T. b. gambiense* and *T. b. rhodesiense*, but not *T. congolense* or *T. vivax* (Giordani et al., 2016). However, it could be surmised that the large bokkosin molecule may not be able to cross the blood brain barrier (BBB) and may therefore only have effect in (early) haemolymphatic stage HAT. A new HAT drug would be most valuable if active against both the late, cerebral stage and the haemolymphatic one, but it should be noted that a non-toxic treatment of early-stage HAT would still be potentially valuable. Until very recently, the recommended treatment for early stage *gambiense* HAT has been pentamidine, since the 1940s, although fexinidazole is now slightly favored for children older than 6 years because of the oral route of administration; both drugs have similar cure rates and levels of adverse effects (Lindner et al., 2020). For early stage *rhodesiense* HAT the treatment has remained suramin since 1920 (de Koning, 2020). Nor can it be assumed *a priori* that bokkosin decidedly will not cross the BBB, as it clearly enters the trypanosome readily enough and, considering its size, this is unlikely to be via a species-specific nutrient transporter as is the case for other trypanocides (Munday et al., 2015). More important yet, bokkosin should now be tested against the dyskinetoplastic trypanosome species *T. evansi* and *T. equiperdum*, which are very closely related to *T. brucei* (Cuypers et al., 2017). These species are the etiological agents of the veterinary conditions surra and dourine, respectively, and are not (necessarily) transmitted by the tsetse fly and thus not limited to sub-Saharan Africa; as such it avoids the potential complication of co-infection with *T. congolense* that would be common in AAT (Giordani et al., 2016).

**Table 6:** EC_50_ of bokkosin against two mammalian cell lines, and the Selectivity Indices for the three kinetoplastid species tested.

**Sample**	**U937**		**RAW 246.7**		**Selectivity Index (S.I) for wild-type**					
	**Mean of EC**_****50****_		**Mean of EC**_****50****_		***T. brucei***		***T. congolense***		***L. mexicana***	
	**(μg/mL)**	**(μM)**	**(μg/mL)**	**(μM)**	**U937**	**RAW**	**U937**	**RAW**	**U937**	**RAW**
Bokkosin	170 ± 7	269	149 ± 5	230	246	215	7.8	6.8	29.5	25.8

*SI, Selectivity Index; IC_50_ (mammalian cell)/EC_50_ (parasite). All EC_50_ values are the average and SEM of three independent determinations*.

## Concluding Remarks

A new compound, bokkosin, was isolated from the traditional medicinal plant *C. portoricensis* and found to be in a novel, unique class. The compound showed promising activity against *T. brucei*, but only moderate activity against *T. congolense* and *Leishmania* species. The trypanocidal activity was almost irreversible even after brief exposure at modest concentrations, while toxicity to mammalian cell types was very low. Thus, bokkosin is a completely new chemical entity, with potent, rapid antitrypanosomal activity against *T. b. brucei* and low toxicity. A logical next step would be the testing of bokkosin against other brucei group trypanosomes such as *T. equiperdum* and *T. evansi*, dyskinetoplastic trypanosomes causing diseases including dourine in Asia, surra in camels in the Middle East, in water buffaloes in southern Asia and horses and dogs in South America (Aregawi et al., 2019).

## Data Availability Statement

The original contributions presented in the study are included in the article/Supplementary Material, further inquiries can be directed to the corresponding author/s.

## Author Contributions

JI, HK, DW, VF, and AG conceptualized the study, supervised the experiments, analyses, and writing of the manuscript. JI, JN, SA, MU, IA, and MN carried out the experiments, data analyses, and contributed to the development of the manuscript. All authors contributed to the article and approved the submitted version.

## Conflict of Interest

The authors declare that the research was conducted in the absence of any commercial or financial relationships that could be construed as a potential conflict of interest.
