# Supplementary material for: Antiparasitic and Cytotoxic Activity of Bokkosin, A Novel Diterpene-Substituted Chromanyl Benzoquinone From Calliandra portoricensis
Source: Front Chem. 2020 Nov 17;8:574103. doi: 10.3389/fchem.2020.574103 (PMC7705231; doi:10.3389/fchem.2020.574103)
Supplement: Supplementary file 1 [file Data_Sheet_1.docx]

**Antiparasitic and cytotoxic activity of bokkosin, a novel diterpene-substituted chromanyl benzoquinone from *Calliandra portoricensis***

**John B. Nvau^1^, Samya Alenezi^2^, Marzuq A. Ungogo^3,4^, Ibrahim A. M. Alfayez^4^,Manal J. Natto^3^Alexander I. Gray^2^, Valerie A. Ferro^2^, Dave G. Watson^2^, Harry P. de Koning^3,^*, John O. Igoli^2,5,*^**

^1^Department of Chemistry, Plateau State University, Bokkos, Nigeria

^2^Strathclyde Institute of Pharmacy and Biomedical Sciences, University of Strathclyde, Glasgow, UK

^3^Institute of Infection, Immunity and Inflammation, College of Medical, Veterinary and Life Sciences, University of Glasgow, UK

^4^Department of Veterinary Pharmacology and Toxicology, Ahmadu Bello University, Zaria, Nigeria

^5^Phytochemistry Research Group, Department of Chemistry, University of Agriculture, Makurdi, Nigeria

**Supplementary materials**

| **No.** | **Primer name** | **Sequence (5’ 🡪 3’)** |
| --- | --- | --- |
| **1** | G00 primer (sgRNA scaffold) | AAAAGCACCGACTCGGTGCCACTTTTTCAAGTTGATAACGGACTAGCCTTATTTTAACTTGCTATTTCTAGCTCTAAAAC |
| **2** | 5' sgRNA primer (*NT1* KO)^1^ | gaaattaatacgactcactataggTGCGACTTTGGATGCACTGAgttttagagctagaaatagc |
| **3** | 3' sgRNA primer (*NT1* KO)^1^ | gaaattaatacgactcactataggACGCATACACAAGCAAGGAGgttttagagctagaaatagc |
| **4** | Upstream forward primer (*NT1* KO)^2^ | TCGCACACATCTCTCGTCCACAAGGCCCCTgtataatgcagacctgctgc |
| **5** | Downstream reverse primer (*NT1* KO)^2^ | GCGATCAACAGCAGTGCGCGGGGCACGCACccaatttgagagacctgtgc |

^1^. 5’ and 3’ sgRNA primers: Lower case indicates the T7 RNA polymerase promoter (left), Upper case indicates the 20 nucleotides of the sgRNA target sequences (middle) and Low case indicates Cas9-backbone-start (right). ^2^. Upstream forward and downstream primers (*NT1* KO): Upper case indicates the 30 nucleotides homology flanks for target gene specific (left) and lower case indicates primer binding sites for pTBlast and pTPuro (right).

**Supplementary Table S1:** The list of primers that were designed and used to generate the NT1KO in *L. mexicana-Cas9*.

**
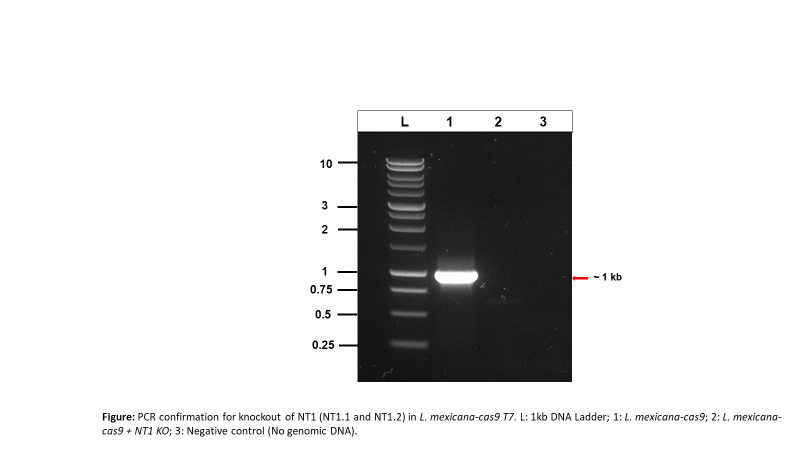
**

**Supplementary Figure 1:** PCR confirmation for the knockout of nucleoside transporter 1 locus (*NT1.1* and *NT1.2* genes) in *L. mexicana-cas9* promastigotes. L: 1kb DNA Ladder; 1: *L. mexicana-cas9;* 2: *L. mexicana-cas9 + NT1 KO*; 3: Negative control (No genomic DNA).

**Supplementary material 1**:High resolution LC-MS for compound **1**.


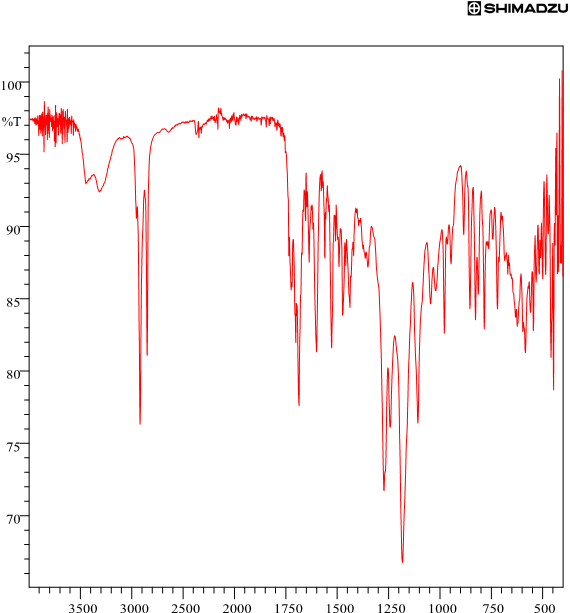


**Supplementary material 2**: IR spectrum for compound **1**

**Supplementary material 3**: ^1^H spectrum for compound **1**in C_6_D_6_

**Supplementary material 4**: ^13^C spectrum for compound **1**in C_6_D_6_

**Supplementary material 5**: COSY spectrum for compound **1**in C_6_D_6_

**Supplementary material 6**: HSQC spectrum for compound **1**in C_6_D_6_

**Supplementary material 7**: HMBC spectrum for compound **1**in C_6_D_6_

**Supplementary material 8**: NOESY spectrum for compound **1**in C_6_D_6_
